# Effects of medium thickness on cellular energy deposition in boron neutron capture therapy

**DOI:** 10.1007/s12194-026-01091-5

**Published:** 2026-07-04

**Authors:** Maheraj Khan, Hiroshi Watabe, Peter K. N. Yu, A. K. F. Haque, M. Ismail Hossain, M. Rafiqul Islam, Gary Tse, Mehrdad Shahmohammadi Beni

**Affiliations:** 1 Department of Physics, University of Rajshahi, Rajshahi, 6205 Bangladesh; 2 Division of Radiation Protection and Nuclear Safety, Research Center for Accelerator and Radioisotope Science, Tohoku University, Sendai, Miyagi 980-8578 Japan; 3 Department of Physics, City University of Hong Kong, Kowloon Tong, Hong Kong, China; 4 Institute of Nuclear Medical Physics, Bangladesh Atomic Energy Commission, Dhaka, 1349 Bangladesh; 5 School of Nursing and Health Sciences, Hong Kong Metropolitan University, Kowloon, Hong Kong, China

**Keywords:** BNCT, Cellular dosimetry, Monte Carlo method, Cell irradiation

## Abstract

Boron Neutron Capture Therapy is dependent on localized energy deposition of alpha particles and lithium nuclei. However, deviations and inconsistencies in cellular responses to neutron beams are frequently reported in radiobiological studies. This technical note investigates dosimetric impact of the overlying medium thickness on a *3× 3* cellular array using Monte Carlo method. $^{10}$B concentrations ranging from 0 to 80 ppm were evaluated to explicitly quantify energy partitioning between the cytoplasm and nucleus. Our findings demonstrate that minor variations in the aqueous medium layer severely attenuate the thermal neutron flux, leading to a marked decrease in the absolute dose deposited in the cellular targets. Specifically, increasing the medium thickness from 100 *μ *m to 800 *μ *m resulted in a $9.8 \pm 1.8\%$ reduction in the total cellular dose at 80 ppm. These results highlight the critical necessity of controlling and reporting fluid levels in BNCT *in vitro* experiments to prevent dosimetric variations and ensure reproducible biological outcomes.

## Introduction

Boron Neutron Capture Therapy (BNCT) is a radiotherapeutic approach relying on the preferential localization of boron-10 ($^{10}$B) enriched compounds within tumor cells, followed by irradiation with low energy thermal or epithermal neutrons [1–3]. The capture of a thermal neutron initiates the $^{10}$B(n,*α *)$^7$Li reaction, yielding high linear energy transfer (LET) alpha (*α *) particles and lithium-7 ($^7$Li) recoil nuclei [4]. The extremely short range of these heavy charged particles in tissue (approximately 5–9 *μ *m) ensures highly localized energy deposition, comparable to the size of a single cell. Consequently, in an ideal case, the BNCT functions as a microscopically selective therapy capable of eradicating boron loaded malignant cells while sparing adjacent healthy tissue.

Considering radiobiological experiments, *in vitro* studies are routinely utilized to evaluate the efficacy of BNCT [5–7]; this is crucial for establishing survival curves for specific cell type. Biological effects of neutrons are relatively less studied and less well understood compared with other types of ionizing radiations such as high-energy photons and heavy ions [8]. Significant variations and deviations in cellular responses to neutron beams are frequently reported across different studies. For instance, research by Wiencke et al. [9] and Ng et al. [10] indicated that neutrons did not trigger a neutron-induced radioadaptive response (RAR) in human lymphocytes or zebrafish embryos. Conversely, Marples and Skov [11] observed a successful neutron-induced RAR in Chinese hamster V79 cells. Further complicating the landscape, Gajendiran et al. [12] analyzed blood samples from ten individuals and found that a neutron-induced RAR was detectable in only a single donor. While these inconsistencies are often attributed to biological variability, a critical yet frequently overlooked physical parameter is the dosimetric influence of the experimental setup itself, specifically, the thickness of the aqueous nutrient medium overlying the cell monolayer.

Previously, we have performed extensive investigations on the effect of medium layer thickness for incident neutrons [8], photons [13], protons [14] and proton boron fusion therapy [15]. It is pertinent to investigate this effect for BNCT. Thermal neutrons exhibit considerable scattering and absorption cross-sections in hydrogen rich media such as water. In a standard culture flask or well plate, cells are submerged under varying amounts of medium, typically ranging from a few hundred micrometers to several millimeters. Because the $^{10}$B neutron capture cross-section is highly sensitive to the neutron energy spectrum, any attenuation or thermalization occurring within this fluid layer can drastically alter the thermal neutron flux that ultimately reaches the target cells. Considering these, the primary objective of this technical note is to quantify the effect of medium layer thickness on the subcellular (cell cytoplasm and nucleus) energy deposition from secondary particles in BNCT using the Particle and Heavy Ion Transport code System (PHITS) [16, 17] Monte Carlo (MC) package; this study underscores the critical importance of the details associated with the irradiation setup in order to achieve reproducible BNCT dosimetry.

## Materials and methods

### Modeled cell array

**Fig. 1:**
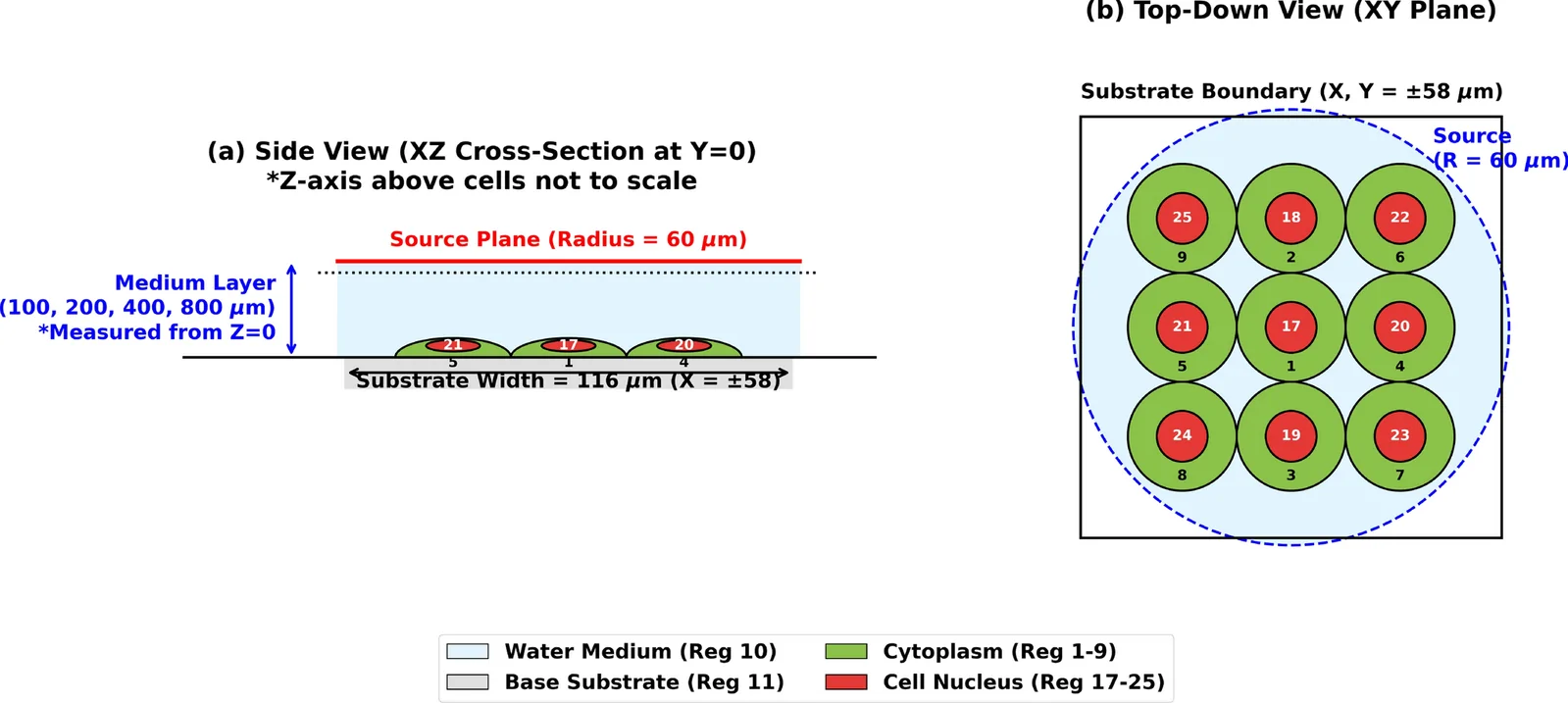
Geometric representation of the *3 × 3* monolayer cell array modeled in PHITS. The side view (left) illustrates the nested nucleus within the flattened semi-ellipsoidal cytoplasm, submerged beneath the medium. The top view (right) shows the tightly packed lattice arrangement. Note: region (Reg) numbers are shown in the cell array

The present model was built using the PHITS MC package version 3.28. The transport and interaction events of different particles were considered, namely, primary neutrons, secondary alpha particles, photons, protons and other recoiled nuclei (defined as nucleus in PHITS tally card). Similar to our previous work [14], an array of cells consisting of nine identical cells with separate cytoplasm and nucleus domains were modeled, see Fig. 1. The nine cells were arranged in a lattice structure and covered by a cylindrical medium (water) layer, with varying thickness of 100, 200, 400, and 800 *μ *m. The modeled cells were half ellipsoids with semi-major, semi-mean and semi-minor axes of 5, 15 and 15 *μ *m, respectively, while the cell nuclei were modelled as ellipsoids with semi-major, semi-mean and semi-minor axes of 1.5, 7 and 7 *μ *m, respectively.

The cell cytoplasm and nucleus were both modeled using the soft tissue composition. The elemental mass fractions consisted of hydrogen (10.5%), carbon (25.6%), nitrogen (2.7%), and oxygen (60.2%), alongside trace amounts of sodium (0.1%), phosphorus (0.2%), sulfur (0.3%), chlorine (0.2%), and potassium (0.2%). While sharing an identical material composition, the cell cytoplasm and nucleus domains were assigned distinct mass densities of $1.03\text { g/cm}^3$ and $1.08\text { g/cm}^3$, respectively. The surrounding medium was modeled as standard water (11.1% H, 88.9% O) with a density of $1.0\text { g/cm}^3$, and the entire geometry was bounded by standard dry air (NIST) at a density of $0.00129\text { g/cm}^3$.

To model semi-ellipsoidal cells, the original full-ellipsoid geometries were bisected using a simulated substrate layer. To prevent the introduction of anomalous physical properties into the model, this substrate was assigned the same material characteristics as the surrounding medium. The substrate acts purely as a geometric cutting plane, defined with an ultra-thin depth of 0.2 *μ *m to slice the cells in half.

### Irradiation setup

**Fig. 2:**
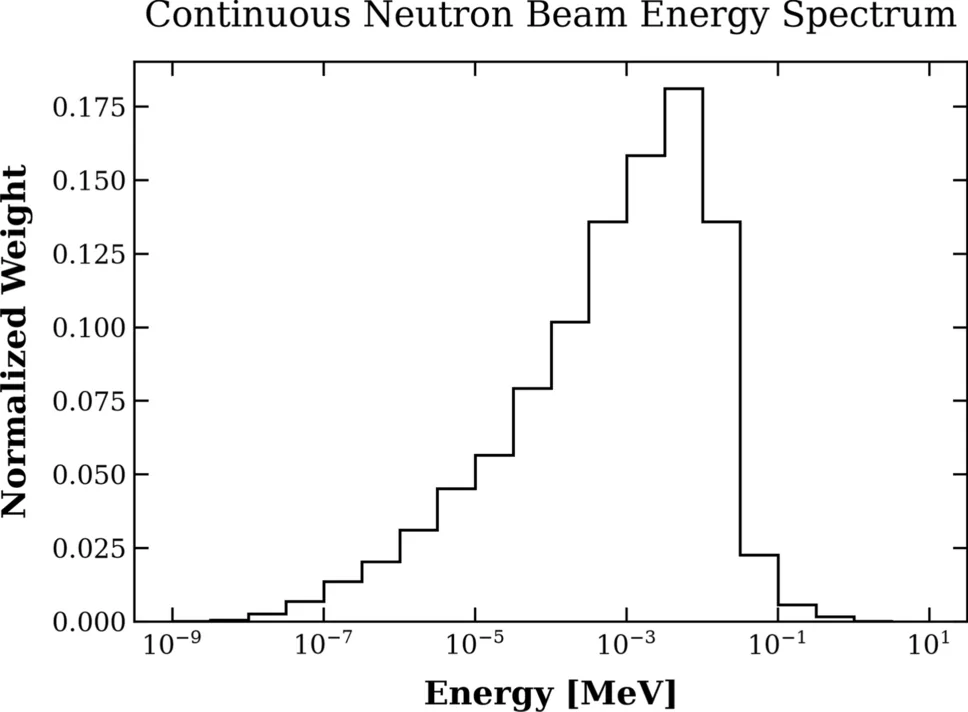
Continuous neutron energy beam used to irradiate the modelled cell array

The radiation source was defined as a planar circular disk with 60 *μ *m radius emitting continuous neutron energy ranging from $\sim 10^{-8}$ to 1 MeV neutrons shown in Fig. 2, directed uniformly downward along the negative *z*-axis. The continuous neutron energy spectrum employed in the present work was constructed based on the general shape and energy bounds reported in Refs. [18–20]. The use of a continuous neutron energy source is highly appropriate for resembling realistic clinical beams. The source was positioned at a very small source-to-target distance of 1 *μ *m above the medium surface. $^{10}$B was homogeneously administered throughout the cytoplasm, nucleus, and the overlying medium. Simulations were conducted across a gradient of $^{10}$B concentrations: 0, 10, 20, 30, 40, 50, 60, 70, and 80 ppm.

The PHITS event generator mode was set to e-mode = 2 for all computations, utilizing Version 2 to ensure accurate charged particle yields and neutron energy distributions. The results presented in our study evaluate the physical dose obtained directly from the MC computations. Furthermore, the thermal neutron scattering law $S(\alpha , \beta )$ was applied to hydrogen atoms in all hydrogenous materials (tissue, nucleus, and water) using the lwtr.20t library that is important for transport of thermal neutrons. All computations were performed using $10^{9}$ primary particle histories via OpenMP on a machine equipped with an AMD Threadripper 9960X CPU utilizing 45 threads.

## Results and discussion

**Fig. 3:**
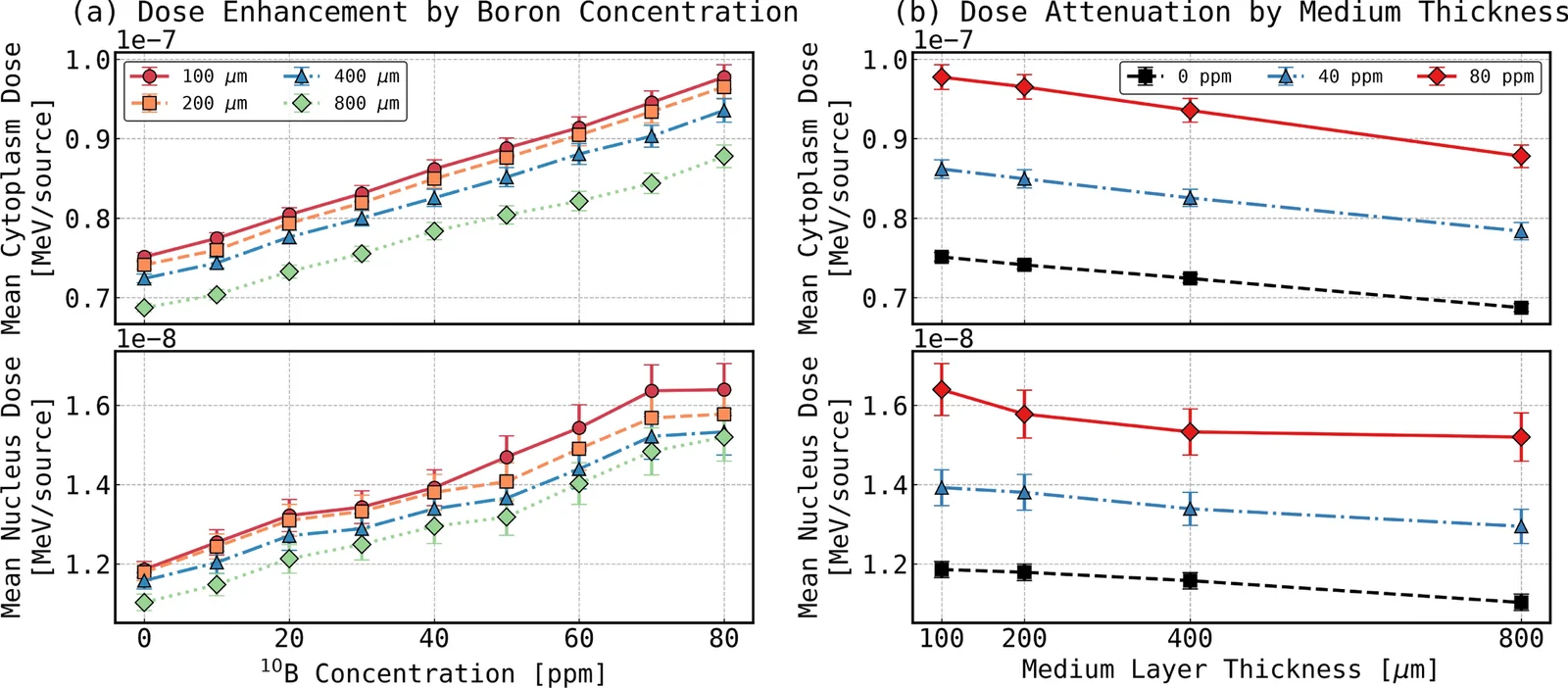
Microdosimetric analysis of **a** dose enhancement by boron concentration for cell cytoplasm and nucleus, **b** dose attenuation by medium thickness for cell cytoplasm and nucleus

**Fig. 4:**
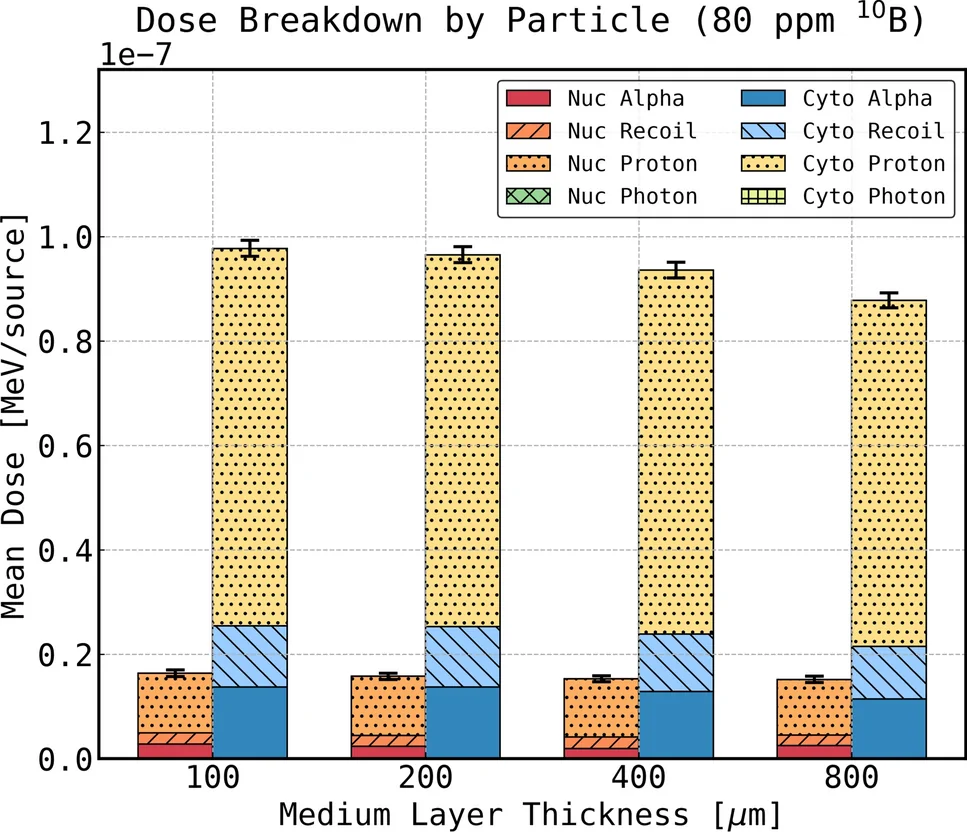
Microdosimetric analysis of dose breakdown by particles at 80 ppm $^{10}$B concentration for 100, 200, 400 and 800 *μ *m medium thicknesses (note: photon dose is negligible in comparison with other particles and therefore cannot be seen in the plot)

**Fig. 5:**
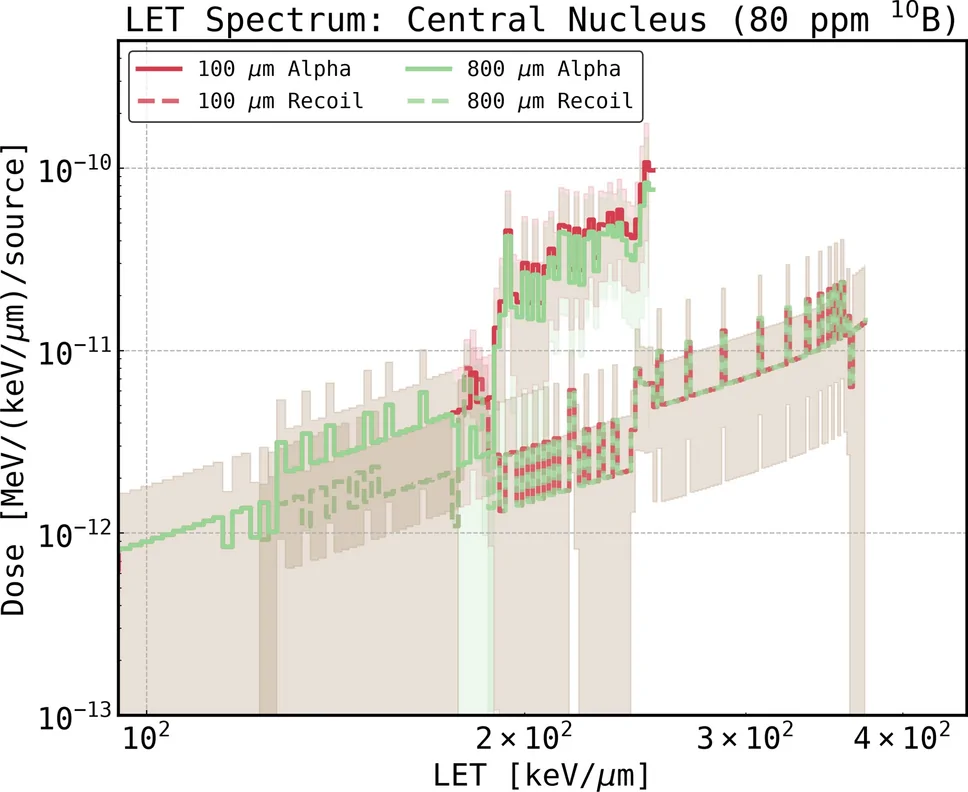
Microdosimetric analysis of LET spectrum at central cell nucleus at 80 ppm $^{10}$B concentration for 100 and 800 *μ *m medium thicknesses

The comprehensive dosimetric results obtained from the present study are illustrated in Figs. 3 to 5. The results shown in Fig. 3(a) demonstrate the dose enhancement driven by varying $^{10}$B concentrations (ranging from 0 to 80 ppm) evaluated across different medium layer thicknesses (100, 200, 400, and 800 *μ *m). The obtained results clearly indicate a proportional increase in the energy deposited within both the cellular cytoplasm and the nucleus as the $^{10}$B concentration rises; this trend is consistent across all evaluated medium thicknesses. The calculated doses incorporate the aggregate effects of alpha particles, recoil nuclei, photons, and protons, with values averaged over the cell cytoplasm and nucleus. The absorbed dose in the cytoplasm was found to be higher than that in the nucleus, a difference primarily attributed to the larger volume of the cytoplasmic compartment.

In addition, Fig. 3(b) illustrates the dose attenuation effect caused by the medium layer overlying the cell array. At specific $^{10}$B concentrations (0, 40, and 80 ppm), an increase in the medium layer thickness results in a measurable, steady reduction of the deposited dose in both cellular compartments. Thicker mediums effectively attenuate the incoming primary radiation flux, thereby lowering the final dose reaching the cell.

A detailed breakdown of the mean dose by contributing secondary particles, specifically at a $^{10}$B concentration of 80 ppm, is provided in Fig. 4. This analysis reveals that the cytoplasm absorbs a substantially higher overall dose compared to the cell nucleus; this is mainly due to the larger cytoplasm interaction volume (base radius 15 *μ *m) compared to the flattened nucleus (radius 7 *μ *m). Primary neutrons will have more interactions with the cytoplasm domain and induce secondary high-LET particles which in turn deposit higher doses in cytoplasm when compared to the cell nucleus domain. The primary contributor to this deposited energy is protons, which account for the vast majority of the dose, followed by significant contributions from alpha particles and recoiled nuclei. In contrast, the dose contribution from photons (predominantly generated *γ *-rays) is found to be minimal to the point of being negligible.

Finally, Fig. 5 illustrates the Linear Energy Transfer (LET) spectrum within the central nucleus (Region 17) for a $^{10}$B concentration of 80 ppm. By isolating the dosimetric contributions of alpha particles and recoil nuclei across medium thicknesses of 100 *μ *m and 800 *μ *m, the spectrum provides a detailed characterization of the radiation quality. A characteristic dual-peak distribution is observed in the high-LET region (approximately $10^2$ to $4\times 10^2$ keV/*μ *m), reflecting the distinct energy deposition profiles of the continuous alpha tracks and the heavier, denser $^7$Li recoil nuclei.

It is crucial to recognize that cell death can be triggered by energy deposited not just in the nucleus, but also within cytoplasmic components. Considering this, the most critical finding of this study is the pronounced attenuation of the total cellular dose as a function of the overlying medium thickness. In order to facilitate direct comparison, the same scale was maintained throughout the plots for different medium thicknesses were used. Considering the optimal 80 ppm $^{10}$B concentrations;

- Beneath a 100 *μ *m medium layer, the total combined dose (alpha + recoil + proton + photon) to the cell constituents (mean dose in cytoplasm + mean dose in nucleus) reaches a sum of ($1.14 \pm 0.02) \times 10^{-7}$ MeV/source.
- Beneath a 200 *μ *m medium layer, this value drops to ($1.12 \pm 0.02) \times 10^{-7}$ MeV/source.
- Beneath a 400 *μ *m medium layer, the dose further degrades to ($1.09 \pm 0.02) \times 10^{-7}$ MeV/source.
- Beneath an 800 *μ *m medium layer, the dose is significantly reduced to ($1.03 \pm 0.02) \times 10^{-7}$ MeV/source.

This represents an approximate $4.6 \pm 1.9\%$ reduction in the absolute total energy deposited in the target cells simply by increasing the water layer from 100 *μ *m to 400 *μ *m, and a $9.8 \pm 1.8\%$ reduction when increased to 800 *μ *m. This is mainly due to the fact that thermal neutrons are highly susceptible to scattering and subsequent capture by hydrogen nuclei ($^1$H(n,*γ *)$^2$H) in the water medium. Consequently, thicker layers of fluid shield the underlying cell monolayer from the primary thermal flux.

In order to capture the complete dose deposited by both alpha particles and $^{7}$Li recoil nuclei, the scoring tally for $^{7}$Li was extended across the entire modeled geometry. At an 80 ppm concentration and an 800 *μ *m medium thickness, the calculated energy deposition ratio was $36.3 \pm 0.5\%$. This finding is in good agreement with the theoretical ratio derived from the respective kinetic energies of the alpha particle (1.47 MeV) and the $^{7}$Li nucleus (0.84 MeV), demonstrating that the simulation correctly models the expected kinematic energy splitting. Notably, restricting the dose scoring exclusively to the cell cytoplasm and nucleus (as reported in this technical note) yields a ratio higher than the theoretical value. This discrepancy primarily arises because alpha particles escape the localized cellular domain where the main results are tallied, and because the restricted tally accounts for all recoil nuclei rather than isolating $^{7}$Li.

## Summary and conclusions

This technical note details a MC investigation into the cellular dosimetry of BNCT, explicitly separating nucleus and cytoplasm energy deposition components across a *3× 3* *in vitro* cell array. The present results confirm that the dose deposition from secondary alpha particles, recoil nuclei, photons and protons in subcellular compartments scales rather linearly with $^{10}$B concentrations.

Crucially, this study highlights an important vulnerability in BNCT radiobiological research and the extreme sensitivity of thermal neutron dosimetry to the overlying medium thickness. We demonstrated that an increase of merely 700 *μ *m in the aqueous layer covering the cells results in a $9.8 \pm 1.8\%$ loss in the delivered dose. In addition, further increasing the medium thickness will lead to a further reduction of the total dose.

Deviations in cellular response and unexpected variations in survival curves reported across different neutron beam facilities may frequently be artifacts of inadequately controlled liquid volumes in culture flasks or well plates. To ensure dosimetric accuracy and biological reproducibility, researchers must strictly measure, standardize, and clearly report the precise fluid thickness used during *in vitro* thermal neutron irradiation scenarios.

## Data Availability

The codes, input parameters and data associated with this study are available from the corresponding author upon reasonable request.
